# Business Methods

**Published:** 1907-12-15

**Authors:** J. H. Irwin


### BUSINESS METHODS.

BY J. H. IRWIN, D.D.S.

In considering the fixing of a fee for a given operation, I find that it is almost impossible to arrive at a satisfactory solution. So long as dentists differ in ability, ambition, avarice, or any other of the many ways which influence the value put upon our services, then so long will the fees remain high with some, medium or low with others. The ambitious dentist constantly compares himself and his surroundings with men more fortunately situated, and strives likewise to increase his skill, practice and fees, so that he may attain and even surpass their position either in the social or the financial world. While the contented, easy-going or timid practitioner compares his lot possibly with the laborer, and finding it easy to out distance him in the race for position or wealth, contents himself with a degree of skill and a recompense far inferior to his energetic brother.

With all these differences of temperament among our professional brothers, it is impossible to expect anything like uniformity in the matter of fees, and it is probably not desirable. But there is one influence in the regulating of our fees which in my opinion is given far too much prominence by many dentists; that is, the character and value of the material used. When the operator allows this matter to be discussed between himself and patient it has a strong tendency to give the proceeding a commercial rather than a professional aspect, and it is to this training that we owe the difficulty of obtaining suitable fees for our time and skill where no material is used. In this matter I constantly try to minimize the commercial value of the material used before my patients. The actual cost of the little pellet of gold, the pinch of alloy cement or porcelain, should never be discussed before the patient in the fixing of a fee, but rather let it be understood that it is the knowledge of where and how to use these materials for their benefit that they are paying. And inasmuch as one material may require greater time and skill for its proper manipulation, your fee must vary for this reason, and not because of the difference of the cost to you.

A set or immovable fee for all operations bearing the same name, regardless of the difference in difficulty and time involved, is in my opinion an injustice to both patient and dentist. Have a minimum fee if you wish, but the less said about it the better. Patients, especially strangers, may want to know, before their work is begun, the likely cost to them of the operation, and although this request is unreasonable to us, they should neither be answered abruptly nor curtly but a little time taken to educate them as to the variety of difficulties and complications liable to be met with in the performances of two operations bearing the same name. In this way our patients may be brought to understand that their dentist should no more be asked beforehand his fee for amalgam fillings, for instance, than a contractor be asked his charge for building bridges or brick houses.

Now, although it is detrimental to the welfare and progress of our profession that dentists should give their services without a respectable fee, it is infinitely worse that so large a number of them should give their time, skill and knowledge for absolutely no recompense whatever. In this class of operations is included extracting free, treating free, advice free and examination free.

In looking at this list of free operations, it would appear that the idea is the offspring of a commercial spirit rather than professional, from the fact that no material passes from the hands of the operator to the patient.

There is no more absurd practice among the many unbusiness-like acts of a dentist than that of extracting teeth for nothing. That a busy dentist will devote the time necessary to removing all the useless teeth from the jaw, with all the unpleasant, even dangerous, associations connected therewith, without fee or reward, is almost incomprehensible. Many a second or third-rate surgeon receives a fee of fifty dollars or more for an operation occupying much less time and skill, but the dentist performs this for the privilege of, six months hence, making a denture for ten dollars, and probably waiting six months more for his pay. This act looks like a bonus for having teeth removed and replaced by artificial substitutes.

The practice of treating teeth free does not appear to be so general, although few among us receive adequate remuneration for the amount of time devoted to preparing unhealthy roots for crowns or fillings; but the cla-s of treatment which is often obtained free from the timid dentist is that given for the relief of pain. The busy man, the careless man or the dead-beat interrupts us, often in the midst of profitable operations, with the request to stick something in his tooth. When that request comes, make a mental determination to get something to "stick in your pocket." This kind of patient never waits quietly for your convenience, but insists on immediate attention. He is usually familiar, and generally calls you "Doc," a name which you deserve if you work for nothing. You leave your profitable patient gurgling behind the rubber-dam, place him in the second chair, and by the time you have your hands washed he has the trouble located very definitely by covering three teeth with the end of his thumb in the region of the second bicuspid. The trouble is usually a long distance from this point. You may find it in the third molar of the opposing jaw. He then begins a fierce argument, and is only brought to respect your opinion by a dash of cold water from your syringe. You treat the trouble, relieve the pain, and as he bends over your cuspidor, not looking you in the face, tells you the fairy story that he is going to have you put all his teeth in good shape. It is then your duty to thank him for his promises and boldly name your fee for the present operation. The dentist who lets that man pay him with "thank you," and a promise, will grow hump-backed, grey-headed and possibly bald before he finds that he is working for dental wholesale houses and greedy landlords. No other class of men are so lax in this regard. For instance, who among you would think of asking your druggist for headache powders, thank him, walk out, and consider him paid when you promise to buy some other drugs when needed.

The dentist is often also imposed upon by those seeking advice. There is a class of people who may dislike you personally and still have great respect for and confidence in your knowledge and ability. These are they who may call upon you, and after an expenditure of considerable time get valuable advice as to what can be done with their teeth, return to their own dentist and dictate to him the knowledge received free from you. From this class of people I invariably demand a fee for advice.

Inasmuch as every community has more people who neglect their teeth than those who attend to them, a possible exception may be made in collecting a fee for the mere examination of teeth, as every encouragement should be given the public to have their teeth examined frequently. The mere examination and telling of the condition need not necessarily include what could or should be done for them.

The second phase of this subject that we have to discuss is the keeping of records. To a dentist of ordinary business instinct this is a matter of prime importance, for if properly done it gives him the useful and interesting information of the amount of work accomplished in a given time, the amount uncollected, the amount of cash received, and the nature of the operations performed for regular patients, year after year. In my own practice I record each name with the amount on a convenient pad, as soon as the patient leaves the chair each day. These names and amounts are entered in a book kept by my office assistant, and the totals for the day, the week and the month prepared so that they can be conveniently seen, for comparison with the total amount received in cash; the cash being kept in a separate book. The difference between the amount of work done and cash received will tell you accurately the amount of uncollected accounts in your books.

In addition to this, I record the work for all regular patients in an "Allport's Dental Register," with the advantages of which the most of you are familiar. By its use the nature, date and fee for the operation can be seen at a glance. It often protects the dentist from being imposed upon by the patient who wilfully or ignorantly misrepresents the facts regarding the operations performed in his mouth. It is also a source of satisfaction to the observant dentist to see how certain materials have stood the test of time.

We have "the fixing of fees," "the recording of fees," and "the collecting of fees;" but the greatest of these is "the collecting of fees," without which the others are as of sounding brass or tinkling cymbal.

No part of our work demands the same amount of courage, tact and knowledge of human nature. I hold that the proper time to think of the collecting of fees is when the patient is taking the chair for the first time. To do justice to your work, your patient and yourself there should be some mutual understanding regarding the circumstances of the patient on one hand, and the approximate fees of the dentist on the other hand. In dealing with your patients regarding this matter they may be divided into three classes. First, your regular patients, including your friends and acquaintances. Second, strangers, who have been recommended to come to you by some former patient. Third, transients, or the people who, seeing your sign, seek your services, not knowing or caring that one dentist exceedeth another in glory. In the first class will be your greatest losses, in the second class your greatest satisfaction, and in the third class, if properly handled, your greatest profit, because, neither knowing dentists nor dentistry, they receive from you for the first time the glad tidings that teeth can be saved. They are not slow to spread the good news, giving your name and methods the full credit; and the dentist who neglects this class of patient, or recklessly extracts teeth that should be saved, is sadly lacking in his full professional duty.

In dealing with my regular patients and acquaintances I follow a rule, without any exceptions, of giving a statement of the amount of their account the day on which their work is finished, before the patient leaves the office, and if a settlement is not made at the time I endeavor to find out what time the patient desires, and make a note of that date on his account. This is given to my office assistant, who calls on him or reminds him through the mail of the time his account is due. This method has the advantage over the one often recommended of sending your account on the first of the following month, in that you have your patient present to explain any little differences by reference to his mouth and your register; but a much greater advantage is that your appreciative patient has not had time to forget your kindly personal interest, the patience and forbearance exercised in his own case, and the tedious and to him monotonous nature of your employment. In this frame of mind a reasonable man will receive and pay your account with a smile, whereas the same amount might produce a howl if sent a few weeks later. Having forgotten much, he might consider himself the victim of a hold-up.

In the case of working for patients for whom another pays, I render an itemized account. In the case of families each child receives a separate and itemized account. This, I know, will not receive the sanction of the ultra-professional among you, but I have yet got to be shown any reasonable objection. The medical profession dces not, the legal profession does, and I think the dental profession may. I do not give the fee for each separate item, but I give the separate dates, and the nature of each operation on that date, and the only fee appearing on the bill is the total, and to this information the parent is entitled.

In dealing with regular patients and acquaintances, eternal vigilance alone will prevent loss, from the fact that friends and acquaintances are apt to impose on your friendship, and former patients who have proved satisfactory in the past in the matter of paying their accounts cannot reasonably be denied credit; but a dentist, without too much parade of money matters, should never lose an opportunity of impressing on his patients the extra trouble incurred in looking after accounts, and the pleasure and time-saving that is afforded by those who settle promptly. An opportunity for a lesson in this matter arises daily in our practices, and we are compelled to leave a patient in a chair and receive payment from another who has probably forgotten his statement, a new account having to be prepared, receipted, and possibly change made. To the patient in the chair it can easily be made apparent that this loss of time could be avoided to our mutual benefit by a settlement when the work was completed. In this matter of paying accounts your patients will treat you very much in the way they have been educated by you. If you are easy-going and unbusiness-like, they will look upon a request for payment as more or less of an insult, and an insinuation that you doubt their honesty. But on the other hand, if you show business ability and system in the carrying out of the various details of your work, both the careless patients and the dishonest ones either mend their ways or give your office a wide berth (a consummation devoutly to be wished.)

The second class are those who have been recommended to your office, and are generally satisfactory from the fact that they have learned your characteristics, the nature of your fees, and, being strangers, will look for no favors. In dealing with this class you are influenced more or less by those who recommended them to come to you; but in my own case, presuming that they are ignorant as to the nature of my fees, and I to their circumstances, I generally suggest a settlement after each sitting, and to this arrangement with this class of patients I can never remember of an objection. The fairness of it to the patient is apparent. It protects him against exorbitant or unknown bills and the dentist from loss.

To the third class, or those seeking your services without any knowledge of you save that given by your sign, there is only one attitude to be taken by any dentist who would rather increase his bank account than his book account.

To those of you who may think that the methods outlined here are too radical for *your* patients, permit me to say, without any idea of boasting, what the results have been in my practice. I began practice fourteen years ago in a town notorious for its credit system, as some in this room can testify. Habits of this kind are forced on communities by necessity. This town was supported years ago by the farmers on one side and the results of the lake industry on the other. Farmers received credit through the summer, and if the Lord was good to them they paid in the winter. Sailors received credit through the winter, and if their lives were spared paid in the summer. With such a system carried on by the best business men, the average dentist had as much chance of prompt settlement as the proverbial snowball; but with careful watching my losses have been small through bad debts. I never sued a delinquent nor employed a collector. In the last few years I have been looking after my practice in the manner here advised, with the result of no falling off in the amount of work done, but a material increase in the amount of cash received. In 1905 my cash receipts were within $50.00 of the total amount of work done, and last year, 1906, the difference was less than $10.00. My patients are better satisfied, and so am I. —*Dominion Journal.*

